# Screening for Chikungunya virus infection in aged people: Development and internal validation of a new score

**DOI:** 10.1371/journal.pone.0181472

**Published:** 2017-08-17

**Authors:** Lidvine Godaert, Seendy Bartholet, Fatiha Najioullah, Maxime Hentzien, Jean-Luc Fanon, Raymond Césaire, Moustapha Dramé

**Affiliations:** 1 Department of Geriatrics, University Hospitals of Martinique, Fort-de-France, Martinique, France; 2 Department of Virology, University Hospitals of Martinique, Fort-de-France, Martinique, France; 3 University Hospital of Reims, Robert Debré Hospital, Department of Infectious Diseases, Reims, France; 4 University of Reims Champagne-Ardenne, Faculty of Medicine, Reims, France; 5 University Hospital of Reims, Robert Debré Hospital, Department of Research and Public Health, Reims, France; Agency for Science, Technology and Research - Singapore Immunology Network, SINGAPORE

## Abstract

**Background:**

This study aimed to derive and validate a score for Chikungunya virus (CHIKV) infection screening in old people admitted to acute care units.

**Methods:**

This study was performed in the Martinique University Hospitals from retrospective cases. Patients were aged 65+, admitted to acute care units for suspected CHIKV infection in 2014, with biological testing using Reverse Transcription Polymerase Chain Reaction (RT-PCR). RT-PCR was used as the gold standard. A screening score was created using adjusted odds ratios of factors associated with positive RT-PCR derived from a multivariable logistic regression model. A ROC curve was used to determine the best cut-off of the score. Bootstrap analysis was used to evaluate its internal validity.

**Results:**

In all, 687 patients were included, 68% with confirmed CHIKV infection, and 32% with laboratory-unconfirmed CHIKV infection. Mean age was 80±8 years, 51% were women. Four variables were found to be independently associated with positive RT-PCR (fever: 3 points; arthralgia of the ankle: 2 points; lymphopenia: 6 points; absence of neutrophil leucocytosis: 10 points). The best cut-off was score ≥12; sensitivity was 87% (83%-90%) and specificity was 70% (63%-76%).

**Conclusion:**

This score shows good diagnostic performance and good internal validation and could be helpful to screen aged people for CHIKV infection.

## Introduction

Chikungunya virus (CHIKV) is an arbovirus transmitted through the bites of infected mosquitoes, predominantly *Aedes aegyptii* and *albopictus*. CHIKV is endemic in tropical regions and has given rise to several epidemics around the world [1–5]. Due to the presence of vectors in temperate regions in Southern Europe and in the Americas, including the United States, the occurrence of epidemics in these naïve regions seems probable [6]. During such epidemic phenomena, prevalence of the disease is high, and can reach up to 70% [7–10]. Several studies have shown that mortality rates increased during CHIK outbreaks [11–16]. Moreover, fatality increases in populations with atypical presentations, and the incidence of such atypical, serious or fatal cases increases with age. Indeed, subjects over 85 years have been shown to be at increased risk of dying [14], and the mortality rate is five times higher in subjects aged 65 years or older (65+) than among those under 45 years of age [11]. On Reunion Island (France), excess mortality from CHIKV concerned mainly people aged 75 years or older (75+) [17]. Therefore, old people who have a symptom profile consistent with CHIKV infection must be diagnosed rapidly.

Management without delay is essential to avoid adverse outcomes. However, establishing a diagnosis of CHIKV infection in a simple and reliable way is very challenging. This concern is especially relevant to the frail aged population. Furthermore, diagnosis based solely on physical examination may underestimate the magnitude of the epidemic [9]. The systematic use of biological diagnosis during an outbreak is not always feasible, especially in low- and middle-income countries [18]. The use of predictive scores would thus be very helpful in this situation. Although there is no specific screening tool dedicated to old patients, they bear the heaviest burden of CHIKV disease. Moreover, Godaert et al [19] recently showed that the only two existing screening tools are not useful in aged subjects. Indeed, the Mayotte tool [20], and the Reunion Island tool [21], which both work well in younger populations, have very poor clinimetric properties in the aged people. Thus, the aim of this study was to develop and validate a new CHIKV screening score specifically for use in the aged population.

## Methods

### Study design and subjects

This study was performed in the University Hospital of Martinique (French West Indies) from retrospective cases. Eligible patients were aged 65 years or older, admitted to acute care units including the emergency department (ED), for suspected CHIKV infection (presence of fever or arthralgia at admission), from 10 January to 31 December 2014, and who underwent biological testing using Reverse Transcription Polymerase Chain Reaction (RT-PCR). Only patients presenting within three days of the onset of symptoms were included. Patients whose clinical and/or biological data were missing in their medical records were excluded.

### Data collection

We recorded baseline characteristics including age, sex, and presence or absence of the following features: fever, arthralgia (any of the following: knee, ankle, metacarpo-phalangeal joints, wrist, elbow, shoulder girdle, and pelvis), low back pain, myalgia, digestive symptoms (nausea, vomiting, diarrhoea…) or neurological symptoms (headaches, space-time disorientation, seizures, neurological deficit, unconsciousness…), and comorbidity burden, assessed using Charlson’s comorbidity index. Biological testing included neutrophils and lymphocyte count, and RT-PCR. Lymphopenia was defined as a count below 1000 lymphocytes per microliter. Neutrophil leukocytosis was considered to be absent if the neutrophil count was below 7500 per microliter. In the acute phase, RT-PCR is considered as the gold standard to identify subjects with or without CHIKV. All patients included in this study underwent serum sample testing using RT-PCR with the RealStar^®^ Chikungunya RT-PCR Kit (Altona Diagnostics GmbH, Hamburg, Germany).

### Ethical considerations

The study was performed in accordance with the Declaration of Helsinki and French legislation relating to research involving human beings. The study was approved by the French national authority for the protection of privacy and personal data (Commission nationale de l’informatique et des libertés, CNIL): authorisation number 1898399 v 0. In French university hospitals, patients are informed in writing that their data contained in their medical records can be used for retrospective research purposes. They have the right to refuse by notifying it orally. If not, once these data are anonymized, the law allows their use with the approval of an ethics committee. This was the case in our study. Indeed, patients’ data was rendered completely anonymous according to the CNIL’s requirements. Data were accessed and analysed retrospectively from the University Hospital of Martinique. The ethics committee of the University Hospital of Martinique granted approval for the study.

### Statistical analysis

Quantitative variables are described as mean±standard deviation (SD), and categorical variables as number (percentage).

Bivariable relationships between each risk factor and CHIK+ status were assessed using logistic regression model with one explanatory variable. Confirmed diagnosis established using RT-PCR was the outcome; baseline characteristics were considered as potential explanatory variables.

Multivariable logistic regression was performed to derive the prediction model. The selection of the candidate variables to be included in the multivariable model was done using a manual stepwise method. The thresholds for entry into and exit from the model were p<0.20 and p<0.10, respectively. Results are presented as odds ratios (OR) and 95% confidence interval (CI).

A point value was assigned to each independent factor according to the adjusted odds ratio of the final model. Point values were rounded to the nearest integer and summed.

The area under the receiver operating characteristics (ROC) curve was calculated (C-statistic), and Hosmer and Lemeshow’s goodness-of-fit test was used to assess the model performance, including discrimination and calibration. Bootstrap analysis was also performed to evaluate the internal validity of the model. Replication on 2000 different samples drawn with replacement was performed using the bootstrap method. The ROC curve was used to determine the cut-off value of the score that best discriminated patients with positive RT-PCR (CHIK+) from patients with negative RT-PCR (CHIK-). The ROC Curve is a plot of the true positive rate (Sensitivity) against the false positive rate (1-Specificity). The cut-off value represents the point on the ROC curve that maximizes both sensitivity and specificity.

The 95% confidence interval (CI) of the C-statistic was also obtained using bootstrap methods. The following diagnostic indices and their 95% CI were computed: Sensitivity, specificity, positive predictive value, negative predictive value. Tests were considered as significant for p-value <0.05. Statistical analyses were performed using SAS version 9.4 (SAS Institute Inc., Cary, NC, USA). The ROC curves were computed using IBM SPSS software release 21 (IBM Corporation, Armonk, NY, USA).

## Results

**Fig 1:**
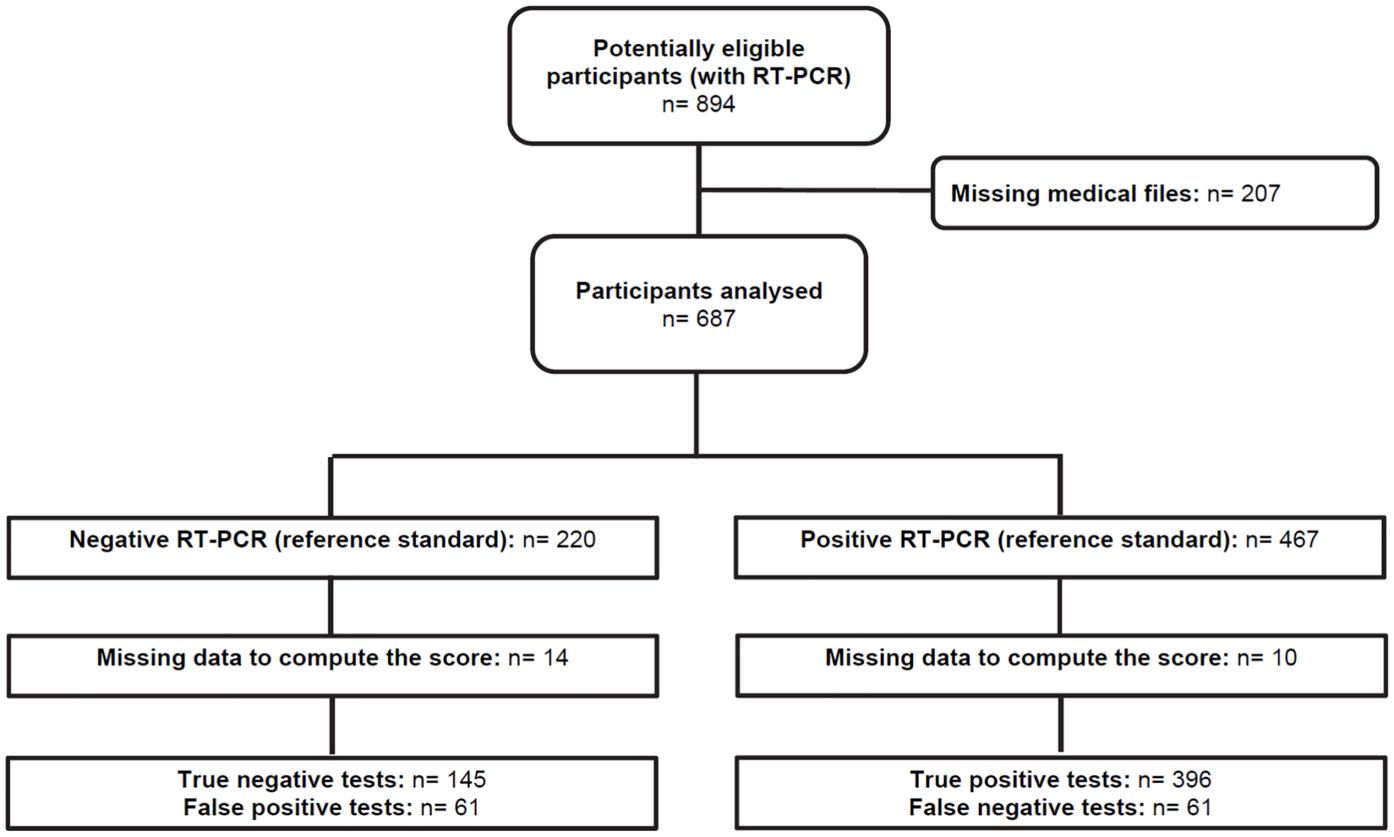
Flowchart of the study population. In all 894 individuals who underwent RT-PCR testing (reference standard) were screened as potential eligible participants. Among them 207 were excluded because of missing files. So 687 were included in the study: 220 had negative RT-PCR and 467 had positive RT-PCR. Among the 220 individuals with negative RT-PCR, 14 had missing data for the score, 145 were classified as negative by both the score and the RT-PCR (true negative), and 61 were classified as positive by the score while being negative with the RT-PCR (false positive). Among the 467 individuals with positive RT-PCR, 10 had missing data for the score, 396 were classified as positive by both the score and the RT-PCR (true positive), and 61 were classified as negative by the score while being positive with the RT-PCR (false negative).

During the epidemic that was ongoing from January to December 2014 in Martinique, 894 patients had RT-PCR for suspected CHIKV in our centre. Among these, 207 were excluded because of missing clinical or biological data. The score was not computed for 24 subjects because of missing data for variables included in the multivariable analysis. A flowchart of the study population is shown in Fig 1. Excluded subjects did not differ significantly from included subjects in terms of age (79.0±8.0 vs. 80.4±8.0 years, respectively) or sex (49% vs. 51% women, respectively).

CHIK+ patients (n = 467) and CHIK- patients (n = 220) included in the analysis did not differ significantly in terms of age (p = 0.33), sex (p = 0.09), or Charlson’s comorbidity score (p = 0.73).

Average lymphocyte and neutrophil cell counts were 1001±719 and 5744±4072 per microliter, respectively. Average Charlson score was 1.7±1.9. Other clinical and biological characteristics at inclusion are presented in Table 1.

**Table 1:** Baseline characteristics of subjects with suspected Chikungunya virus infection.

Symptoms or association of symptoms	Total N = 687		CHIK+ n = 467		CHIK− n = 220		
	n	%	n	%	n	%	p*
Fever	502	73.1	371	79.4	131	59.6	< .0001
Arthralgia	353	51.4	292	62.5	61	27.7	< .0001
Arthralgia of the wrist	87	12.7	75	16.1	12	5.5	< .0001
Arthralgia of the metacarpophalangeal joints	47	6.8	40	8.6	7	3.2	.009
Arthralgia of the ankle	131	19.1	110	23.6	21	9.6	< .0001
Arthralgia of the knee	153	22.3	127	27.2	26	11.8	< .0001
Arthralgia of the shoulder	58	8.4	49	10.5	9	4.1	.005
Arthralgia of the pelvis	25	3.6	22	4.7	3	1.4	.03
Low back pain	69	10.0	48	10.3	21	9.6	.77
Myalgia	114	16.6	82	17.8	32	14.6	.32
Digestive symptoms	151	22.0	91	19.5	60	27.3	.02
Neurological symptoms	258	37.6	166	35.6	92	41.8	.11
Lymphopenia	407	61.3	345	75.3	62	30.1	< .0001
Absence of neutrophil leukocytosis	517	77.9	415	90.6	102	49.5	< .0001

Missing data: neutrophils (n = 23); lymphocytes (n = 23)—CHIK+: Positive RT-PCR; CHIK-: Negative RT-PCR–

* Pearson’s Chi2 test

By multivariable analysis (Table 2), four variables were found to be independently associated with CHIK+ status, namely fever, arthralgia of the ankle, lymphopenia, and absence of neutrophil leukocytosis. Bootstrap methods showed good internal validity of the final model.

**Table 2:** Bivariable and multivariable analyses of predictors of CHIK+ status using logistic regression, and the corresponding weighted point values of the score.

	Bivariable analysis			Multivariable analysis			Point value
	OR	95% CI	p	OR	95% CI	p	
Fever^#^	2.6	1.8–3.7	< .0001	2.9	1.9–4.6	< .0001	3
Arthralgia	4.3	3.0–6.1	< .0001				
Arthralgia of the wrist	3.3	1.8–6.2	.0002				
Arthralgia of the metacarpophalangeal joints	2.8	1.3–6.5	< .0001				
Arthralgia of the ankle^#^	2.9	1.8–4.8	< .0001	2.3	1.2–4.1	.008	2
Arthralgia of the knee	2.8	1.8–4.4	< .0001				
Arthralgia of the shoulder	2.7	1.3–5.7	.007				
Arthralgia of the pelvis	3.6	1.1–12.1	.04				
Low back pain	1.1	0.6–1.9	.77				
Myalgia	1.2	0.8–1.9	.32				
Digestive symptoms	0.6	0.4–0.9	.02				
Neurological symptoms	1.0	0.6–1.5	.94				
Lymphopenia^#^	7.1	4.9–10.2	< .0001	6.0	3.9–9.1	< .0001	6
Absence of neutrophil leukocytosis^#^	9.8	6.5–14.9	< .0001	10.4	6.4–16.9	< .0001	10

OR: Odds Ratio; CI: Confidence Interval

^#^ Variables retained in the final multivariable analysis, and therefore, included as components of the score. Point values assigned to each variable included in the score are indicated in the right hand column of the table.

The point values assigned to each of the predictive factors identified in the final model are listed in Table 2.

**Fig 2:**
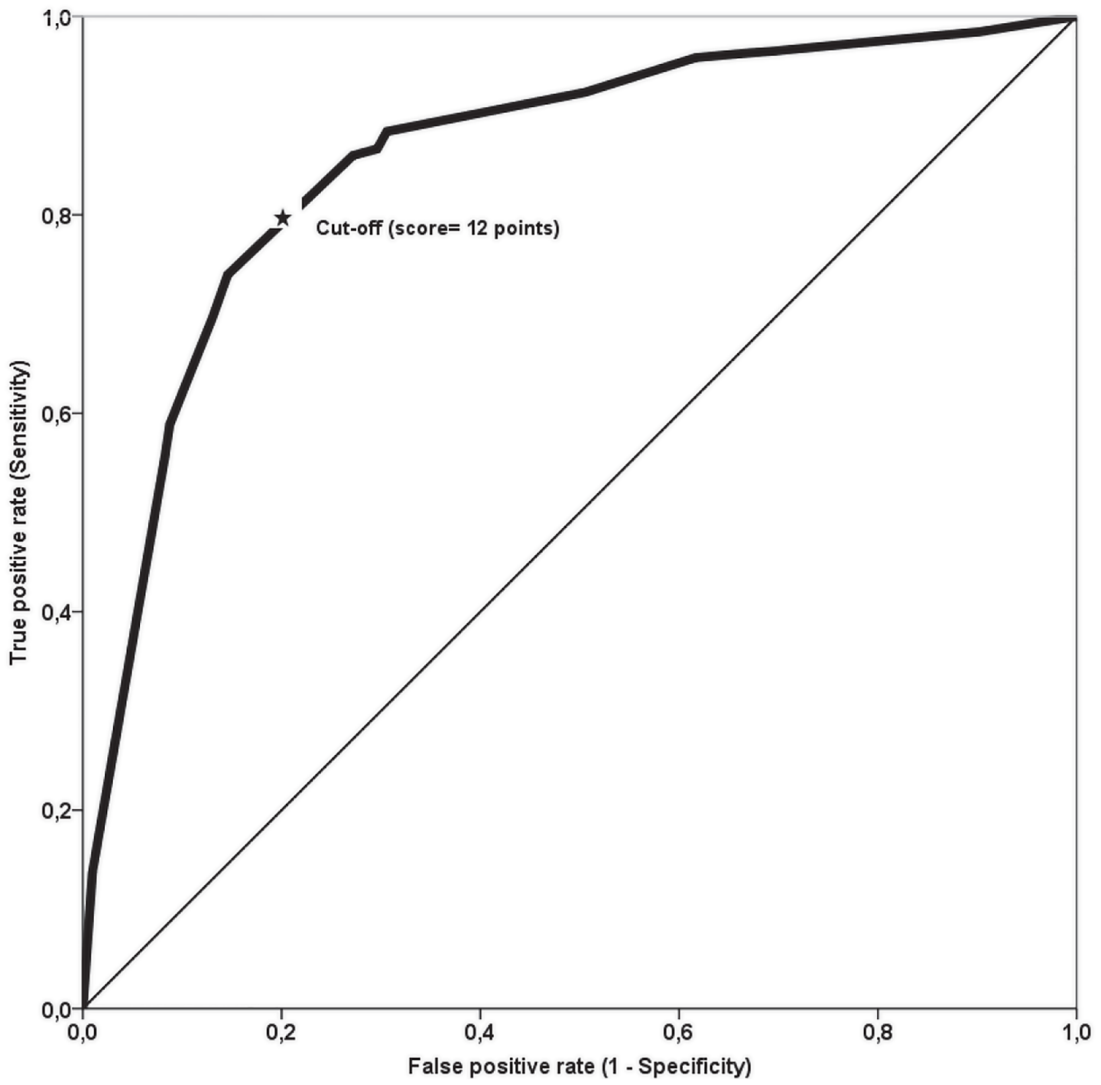
Receiver operating characteristic of the screening score for Chikungunya virus infection. The Receiver Operating Characteristic (ROC) Curve is a plot of the true positive rate (Sensitivity) against the false positive rate (1-Specificity). The cut-off value represents the point on the curve that maximizes both sensitivity and specificity. In this analysis, the cut-off value identified to best distinguish between CHIK+ and CHIK- patients was 12 points (★).

Scores ranged from 0 to 21, with an average of 14±6. Fig 2 presents the ROC curve. The uncorrected C-statistic was the same as the bootstrapped C-statistic, namely 0.86 (95% CI: 0.83–0.89). The ROC curve identified the cut-off value for the score that maximized both sensitivity and specificity. A score greater or equal 12 was considered as the best cut-off to identify CHIK+ patients, with a sensitivity of 87% (95% CI: 83%-90%) and a specificity of 70% (95% CI: 63%-76%). Other diagnostic indices are presented in Table 3.

**Table 3:** Diagnostic performances of the score dichotomised at 12 points*.

	Value	95% CI
Sensitivity	87%	83%– 89%
Specificity	70%	64%– 76%
Positive predictive value	87%	83%– 89%
Negative predictive value	70%	64%– 76%

* RT-PCR was considered as the gold standard

Missing values precluding calculation of the score: n = 24

## Discussion

We developed and validated a score to screen for CHIKV in old patients. The score includes four variables (fever, arthralgia of the ankle, lymphopenia, and absence of neutrophil leukocytosis) identified using multivariable logistic regression. These variables are independently associated with positive RT-PCR status, and are used in routine practice.

Fever is a common sign of CHIKV infection, along with polyarthralgia, headaches and macular rush [2, 21, 22]. Painful complaints as a sign in CHIKV are difficult to establish in aged population. Although polyarthralgia is reported in 87% to 98% of cases in young adults [22], and joint pain occurs mainly in peripheral joints such as the ankle or wrist [23], in our study only six in ten persons with CHIKV reported having arthralgia. There are two possible explanations for this observation. Firstly, either old people underreport pain because of its chronicity, or secondly, the manifestations of acute illness differ between older and younger adults. Indeed, even when pain is frequent, it is understated in aged populations [24, 25]. In typical acute CHIKV infection, arthritis or arthralgia of the ankle is described [22, 23, 26, 27], and indeed, in our study, arthralgia of ankle was found to be an independent predictive factor of positive RT-PCR. In aged people, this sign appears to be more characteristic than other localisations, such as the knee [28, 29]. In typical CHIKV presentations, no neutrophil leukocytosis observed, and lymphopenia is frequently reported [21, 22]. As far as we know, no study to date has reported other specific biological signs in older people, and there is no study to date about the clinical syndrome of CHIKV in acute illness in older people.

The score developed and validated here is the first that is specifically dedicated to the aged population. It was derived using usual and accurate statistical methods. Moreover, its internal validation was very good. Its accuracy in old people is better than that of scores developed in younger populations [20, 21]. Indeed, we recently showed [19] that the two existing scores have very poor clinimetric performances in aged people. When using these two scores to screen for CHIKV in the elderly, sensitivity ranged from 6% to only 49%, and Youden’s statistic ranged from 1% to 30%. These poor performances thus prompted us to develop a new score, more suitable for old patients. Our score specifically targets older patients, who may have different clinical symptom profiles than younger subjects.

Among the strengths of our study, we can underline the fact that RT-PCR was used to ascertain the diagnosis of CHIKV. Laboratory diagnosis relies on the detection of the virus on early samples (day 1 to day 7) and/or specific anti-Chik IgM and/or IgG in blood samples (after day 5). Commercial Chikungunya RT-PCR kits are available with excellent diagnostic performances [27], and RT-PCR is recommended for early diagnosis [30]. In our study, all the subjects included underwent RT-PCR serum testing in the same laboratory. The accuracy of our score was very good (area under the ROC curve = 0.86), and remained unchanged between iterations of internal validation using the bootstrap method. This is probably due to the large sample used to derive it. This tool is thus potentially relevant as an alternative to CHIKV laboratory testing, mainly during outbreaks. Indeed, as stated by Sissoko et al [20], in the context of an outbreak, the time and resources required for compulsory laboratory confirmation are lacking. Hence, our score is potentially useful, as it is derived from easy-to-access data (clinical features, and blood count). Even in low income countries, blood counts are increasingly performed in routine practice. The score is thus easy to calculate by simply summing the different point values assigned to the four predictors. A cut-off established at 12 points or more enables the identification of CHIK+ subjects, with good accuracy, and good pre-test (sensitivity and specificity), and post-test (PPV, PNV) performances. We therefore believe that this score could be widely used.

Conversely, we acknowledge that the main limitation relates to the absence of external validation. However, as outbreaks are rare, as are geriatric care units involved in CHIKV research, it is very challenging to constitute a validation cohort.

In conclusion, our results show that a score based on easy-to-access clinical and biological features obtained in routine practice, has good discrimination and calibration properties in aged population. It could be a relevant alternative to laboratory testing in the context of CHIK outbreaks, or in settings with limited resources.
